# Evaluation of the Comparative Efficacy of Navak Guggulu Versus Vachaharidradi Gana Vati in the Management of Sthaulya (Overweight) in Children: Protocol for a Randomized Controlled Trial

**DOI:** 10.2196/58838

**Published:** 2025-10-07

**Authors:** Kavita Kishor Fadnavis, Renu Bharat Rathi, Karuna Sunil Ratnaparkhi, Bharat Jagdishji Rathi, Paresh Rameshrao Deshmukh

**Affiliations:** 1 Department of Kaumarbhritya Mahatma Gandhi Ayurved College Hospital and Research Centre Wardha India; 2 Department of Kaumarbhritya Chhatrapati Shahu Maharaj Shikshan Sanstha's Ayurved College and Hospital Chhatrapati Sambhajinagar India; 3 Department of Rasa Shastra and Bhaishajya Kalpana Mahatma Gandhi Ayurveda College Hospital and Research Centre Wardha India; 4 Department of Kayachiktsa Chhatrapati Shahu Maharaj Shikshan Sanstha's Ayurved College and Hospital Chhatrapati Sambhajinagar India

**Keywords:** childhood obesity, Sthaulya, Vachaharidradi Gana Vati, Navak Guggulu, Sun Salutation

## Abstract

**Background:**

Childhood obesity is a lifestyle-oriented disorder, which may lead to many comorbidities such as hypertension, coronary diseases, increased incidence of diabetes, and metabolic syndromes. Lack of diet control and increased screen time specifically after the COVID-19 pandemic among children and adolescents are the major reasons for this rising global concern. Despite various campaigns being carried out to lessen the rising rates of childhood obesity, the objectives have not yet been achieved. Ayurveda offers a multidimensional modality in managing children with overweight. The purpose of this study is to evaluate the combined efficacy of Ayurvedic medicine administration, diet restriction, and Sun Salutation. Navak Guggulu is a herbo-mineral compound already proven for its antiobesity action. Vachaharidradi Gana mentioned in Ayurvedic classics is a group of herbs, which are Kaphamedohara and Karshana. Ayurveda also emphasizes the importance of diet and physical activities for the treatment of obesity. The interventions together may help in promoting a healthy weight, improving metabolism, and reducing fat accumulation. So, in this study, the efficacy of Vachaharidradi Gana Vati will be compared with Navak Guggulu to treat children with overweight in addition to diet restrictions and Sun Salutation.

**Objective:**

The aim of the study is to evaluate the efficacy of Navak Guggulu versus Vachaharidradi Gana Vati along with diet restriction and Sun Salutation in overweight (Sthaulya) children.

**Methods:**

A double-blind randomized interventional study will be conducted on selected children with overweight in the age group of 10 to 16 years. They will be equally distributed into 2 groups. The control group will be given Navak Guggulu, and the trial group will be given Vachaharidradi Gana Vati for 90 days. Both groups will be advised of diet restrictions and the daily performance of the Sun Salutation. The study duration is 180 days, with assessment at baseline, followed by every 15 days up to 90 days, and at the end point, that is, after 180 days. Appropriate statistical techniques, such as analysis of covariance paired 2-tailed *t* test, Wilcoxon signed rank test, Mann-Whitney *U* test, and multivariate regression analysis, will be used to evaluate the efficacy of the interventions.

**Results:**

The study got underway in April 2024. Since participants may begin at any time, our goal is for everyone to be finished by May 2025.

**Conclusions:**

This research will be useful to provide new insight into the effectiveness of the Ayurveda pharmacological approach in treating obesity in children. The results will guide evidence-based multimodality in treatments aimed at changing lifestyle behaviors in children with obesity, thus reducing social stigmatization in them.

**Trial Registration:**

Clinical Trial Registry of India CTRI/2023/03/051135; https://ctri.nic.in/Clinicaltrials/pmaindet2.php?EncHid=NzgzNzE=&Enc=&userName=

**International Registered Report Identifier (IRRID):**

DERR1-10.2196/58838

## Introduction

### Background

India is going through a socioeconomic, nutritional, cultural, and health transformation. In recent years, the country has been witnessing an increase in the overnutrition rates in children. It shows the emergence of the dual nutrition burden in India, namely, overnutrition and undernutrition. Overnutrition among children manifests in overweight or obesity. Children from affluent urban families are more prone to obesity; however, recent data indicate a significant rise in obesity rates among low-income groups in both rural and urban areas. Globalization has introduced multinational fast-food chains across India, leading to a shift from traditional to modern eating habits. This transition has resulted in increased consumption of processed, high-calorie, ready-made foods, particularly among children. Such foods, rich in sugars and salts, have significantly influenced dietary preferences [1-4].

According to the International Obesity Task Force categorization in 2013, the overall prevalence of overweight and obesity was 18.2% and 23.9%, respectively, as per World Health Organization (WHO) standards [5]. As per computation by Lobstein and Jackson-Leach [6], we expect the number of children with obesity in India to reach 17 million by the end of 2025. In a systematic review, the pooled data from the states of India after 2010, the combined prevalence of childhood overweight and obesity was predicted to be 19.3% [7]. The COVID-19 pandemic situation further added a load to it [8]. During the lockdown, web-based schooling forced children to stay indoors, which reduced their physical activity and increased their screen time, leading to prolonged sitting with increased screen time. Excessive screen time reduces physical activity, which results in fat deposition at an early age [9].

Overweight or obesity is characterized by increased weight, increased weight for height, and raised BMI [10]. Recently, new Indian standards have been notified, and new 5- to 17-year charts of BMI for boys and girls have been published by the Indian Academy of Pediatrics. According to that, the BMI cutoffs for overweight and obesity in children are defined at 23 and 27 kg/m^2^, respectively (Indian Academy of Pediatrics 2015) [11]. Obesity is a risk factor for numerous noncommunicable diseases, including type 2 diabetes mellitus, asthma, joint pain, and multiple health conditions [12-16]. It frequently carries a high risk of early mortality and significant disability [17]. Children with overweight are more likely to experience cognitive developmental issues and are more likely to become adults with obesity. Obesity prevention should begin in childhood. According to the WHO expert constitution, on average, obesity reduces life expectancy by 6-7 years in adults [18-20].

Despite various campaigns being carried out to lessen the rising rates of childhood obesity, the objectives have not been achieved. The main causes include lack of awareness, poor adherence to balanced diet guidelines, and the absence of comprehensive policies to address this hidden epidemic [21]. It has become the need of the hour to recognize children with overweight and treat them as early as possible.

According to Ayurvedic classics, Sthaulya can be correlated with overweight or obesity. It is a Santarpanajanya Vyadhi [22], which means the disease caused due to excessive intake of food and decreased physical activities. In Ayurveda, the management of Santarpanajanya Vyadhi is a multidimensional modality. It includes medicine administration, Nidanaparivarjana [23] (avoidance of causative factors), diet control, and Vyayama (physical exercise). Ayurveda offers various medicines for the treatment of Sthaulya. Navak Guggulu is a well-known polyherbal compound containing guggulu (*Commiphora mukul* Linn) as the main ingredient. This compound is reported for its antiobesity action [24-27]. It has also been studied in the management of hyperlipidemia [28]. Though it is a proven drug, the availability of pure guggulu is a difficult task day by day. There is a scarcity of pure guggulu, and it is a tedious job to purify guggulu for oral administration, as the available guggulu contains many impurities.

Vachaharidradi Gana mentioned in Ayurvedic classics is a group of herbal medicines that have properties of Kaphamedohara (alleviating Kapha and reducing fats) and Karshana (therapeutic scraping). These are the characteristics of the medications that are recommended in the management of Santarpanajanya Vyadhi like overweight and obesity to improve fat metabolism. As a result, it should aid in maintaining weight and BMI. Further, the contents of Vachaharidradi Gana are easily available and cost-effective as well.

Diet control is as important as medicines in the management of Sthaulya. Bad eating habits are the main cause of obesity among children [29]. Eating in front of the television screen, drinking sugar-sweetened beverages, eating junk foods and packaged foods, and eating without hunger are the factors responsible for gaining excessive weight. Ayurveda has also emphasized the importance of diet in one’s health by phrasing it as “Mahabheshaja,” which means the most important medicine [30]. Therefore, strict diet control must be advocated for the management of children with overweight.

Physical activity is also important for preventing and managing obesity. Lack of exercise and obesity go hand in hand. Increasing physical activity in children is a nonrestrictive approach to obesity management, which is the discretionary component of energy expenditure [31,32]. Suryanamaskara (Sun Salutation) mentioned by Sayanacharya [33] is an ancient, Vedic, and sacred yogic technique that evolved in India for expressing gratitude to the Lord Sun. It is a series of asanas (postures). It helps in the activation of fat metabolism by rigorous movements and ultimately helps in the reduction of fat, thus beneficial in BMI reduction [34]. It also helps in psychological well-being [35], which will lead to a decrease in screen time.

If children with overweight are educated enough about diet restriction and performing Suryanamaskara along with Ayurvedic remedies, it will be beneficial to them to lead a healthy and happy life.

### Objectives

The aim of the study is to evaluate the equivalent efficacy of Navak Guggulu versus Vachaharidradi Gana Vati along with diet restriction and Suryanamaskara in children with Sthaulya (Overweight).

#### Primary Objectives

The primary objectives are (1) to evaluate the clinical efficacy of Navak Guggulu along with diet restrictions and Suryanamaskara in children with Sthaulya (overweight) on BMI and other parameters, (2) to evaluate the clinical efficacy of Vachaharidradi Gana Vati along with diet restrictions and Suryanamaskara in children with Sthaulya (overweight) on BMI and other parameters, and (3) to compare the efficacy of Navak Guggulu and Vachaharidradi Gana Vati along with diet restrictions and Suryanamaskara as adjuvant therapies in children with Sthaulya (overweight) on BMI and other parameters.

#### Secondary Objectives

The secondary objectives are (1) to evaluate the residual efficacy of Navak Guggulu versus Vachaharidradi Gana Vati in children with Sthaulya (overweight) after discontinuation of treatment after 180 days as well; (2) to study the effect of desha, kala, and prakruti on the children with overweight; and (3) to study the adverse effects of Vachaharidradi Gana Vati if any.

### Hypotheses

The hypotheses are as follows:

- H0 null hypothesis: Vachaharidradi Gana Vati and Navak Guggulu along with diet restrictions and Suryanamaskara are not equivalent in efficacy in reducing the BMI in children.
- H1 alternate hypothesis: The efficacy of Vachaharidradi Gana Vati is equal to Navak Guggulu along with diet restrictions and Suryanamaskara in reducing the BMI in children.

## Methods

### Study Design

**Figure 1:**
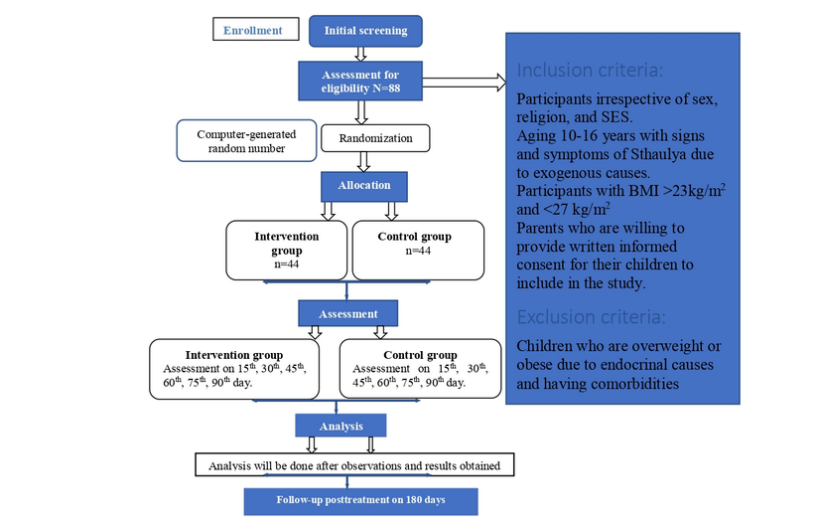
The study design. SES: socioeconomic status.

A randomized, standard-controlled, double-blind equivalence clinical trial will be performed. The study flow is depicted in Figure 1. The study participants required for the trial include children with overweight aged between 10 and 16 years of age will be selected from the Outpatient Department and Inpatient Department of the Department of Kaumarbhritya, Mahatma Gandhi Ayurveda College Hospital and Research Centre, Salod (H), and Chhatrapati Shahu Maharaj Shikshan Sanstha’s (CSMSS) Ayurved College and Hospital, Chhatrapati Sambhajinagar, based on the inclusion criteria.

Overweight (Sthaulya) children will be diagnosed as having a BMI greater than or equal to 23 and less than 27 kg/m^2^ [11]. Screening will include hemoglobin percentage, thyroid-stimulating hormone, and random blood sugar. A total of 88 diagnosed children with overweight will be selected after obtaining written informed consent from their guardians. Participants will be randomly divided into 2 groups by block randomization using a computerized random number generator or statistical software. Group A (control group) will receive Navak Guggulu, while group B (trial group) will receive Vachaharidradi Gana Vati orally (Table 1). Medications will be dispensed in opaque containers following the sequentially numbered, opaque, sealed envelope (SNOSE) technique to maintain allocation concealment. Both groups will receive counseling to perform 12 Suryanamaskara (Sun Salutation) per day and adhere to the dietary restrictions. Guardians will be educated and advised to support their children’s consistent participation in the interventions. All the baseline parameters will be recorded at the study’s initiation. The treatment duration will be 90 days, with follow-up assessments on the 15th, 30th, 45th, 60th, 75th, and 90th day to monitor progress and ensure adherence. Lipid profile will be assessed before treatment and on the 90th day of intervention to evaluate the efficacy of the interventions. An additional follow-up will occur on the 180th day to determine whether the intervention’s possible effects are lasting.

**Table 1:** Overview of intervention.

	A (control group)	B (intervention group)
Participants, n	44	44
Name of medication	Navak Guggulu	Vachaharidradi Gana Vati
Dosage	Young’s formula	Young’s formula
Frequency	Thrice a day before meals	Thrice a day before meals
Duration	90 days	90 days
Assessment	1st, 15th, 30th, 45th, 60th, 75th, and 90th day	1st, 15th, 30th, 45th, 60th, 75th, and 90th day
Follow-up	180th day	180th day
Route of administration	Oral	Oral
Anupana	Lukewarm water	Lukewarm water

The Suryanamaskara techniques following the Bihar School of Yoga Tradition will be advocated to the children [36,37]. Each participant will be provided with detailed written instructions, provided in local language, outlining the Ayurvedic diet restrictions as follows: avoid eating all sweet food items (chocolate, ice creams, etc), potatoes, dairy products (paneer, curd, cheese, Shrikhand, etc), fried foods (wafers, puri, and pakora), and bakery products (biscuits, pastries, cakes, bread, etc); refrain from consuming junk foods and fast-food items; avoid sweetened soft drinks and beverages; and avoid overeating and eating without hunger and ensure appropriate intervals between meals. Patients will be given regular reminders also through calls or SMS text messages to follow diet restrictions and perform Suryanamaskara. SPIRIT (Standard Protocol Items: Recommendation for Interventional Trials) guidelines will be used for the study.

### Inclusion Criteria

The inclusion criteria are as follows: participants irrespective of gender, religion, and socioeconomic status, age group between 10 and 16 years with signs and symptoms of overweight (Sthaulya) due to exogenous causes, participants with BMI ≥23 and <27 kg/m^2^, and parents who are willing to provide written informed consent for their children to be included in the study.

### Exclusion Criteria

The exclusion criteria are as follows: children having BMI <23 and ≥27 kg/m^2^, children aged <10 years and those aged >16 years, and children who are overweight due to endocrine causes. Children experiencing severe systemic illnesses like hypertension, asthma, diabetes mellitus, nephrotic syndrome, kwashiorkor, and congestive cardiac failure having anasarca condition are overviewed as overweight.

### Drug Collection or Authentication

The raw material for the both the control and intervention drugs will be purchased from a reliable source and will be authenticated and identified by the department of Dravyaguna and Rasashastra of Mahatma Gandhi Ayurved College Hospital and Research Centre, Salod, Wardha.

### Details of Drug Composition and Preparation Method

The control drug is Navak Guggulu, which is a polyherbal compound mentioned in Yogaratnakara [38]. It has been proven to have antiobesity effects in many randomized controlled trials (RCTs). The composition of Navak Guggulu is presented in Table 2.

**Table 2:** Latin name and family name of the herbs used in Navak Guggulu.

Serial No	Drug	Latin name	Family
1	Shunthi	*Zingiber officinale*, Roscoe	Zingiberaceae
2	Pippali	*Piper longum*, Linn	Piperaceae
3	Marich	*Piper nigrum*, Linn	Piperaceae
4	Haritaki	*Terminalia chebula*, Retz	Combretaceae
5	Bibhitaki	*Terminalia bellirica*, Roxb	Combretaceae
6	Amalaki	*Emblica officinalis*, *Phyllanthus emblica*, Linn	Euphorbiaceae
7	Musta	*Cyperus rotundus*	Cyperaceae
8	Chitrak	*Plumbago zeylanica*, Linn	Plumbaginaceae
9	Vidanga	*Embelia ribes*, Burm	Myrsinaceae
10	Guggulu	*Commiphora mukul* or *Balsamodendron mukul*, Hook	Burseraceae

### Preparation Method of Navak Guggulu

In the Good Manufacturing Practice (GMP)–certified pharma company, guggulu will be first purified by dissolving in Triphala kwatha (decoction), and then, the decoction will be filtered and concentrated by heating it. In that concentration, the remaining herbs in the form of fine powder will be mixed well. Then, this mass will be dried in the drying machine. With the help of a punching machine, tablets of 300 mg will be made. Each tablet of Navak Guggulu approximately contains 150 mg of pure guggulu and 16.66 mg of remaining herbs each.

**Figure 2:**
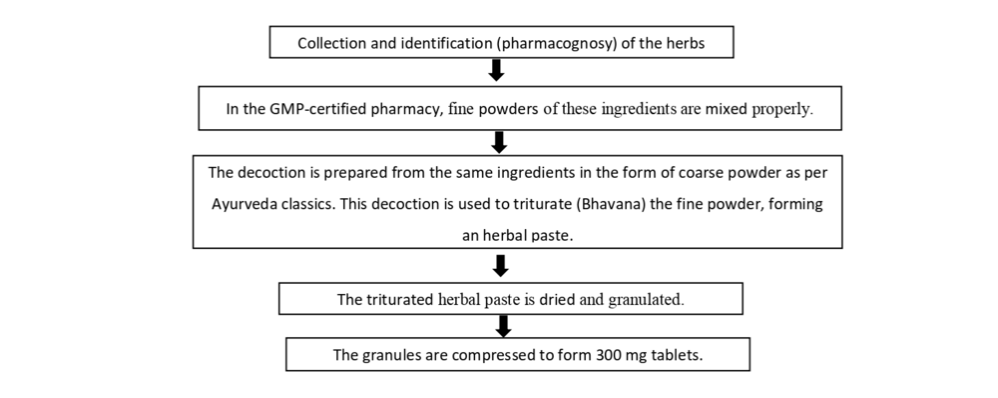
Preparation method of Vachaharidradi Gana Vati. GMP: Good Manufacturing Practice.

The intervention drug is Vachaharidradi Gana mentioned by Acharya Vagbhata in his classic Ashtanga Hridaya [39]. It has the opposite qualities to Kapha and Meda. Keeping this fact in mind, selected herbs from Vachaharidradi Gana were selected for oral use in children with overweight in the modified form of tablet (vati). The selected drugs are detailed in Table 3, and their preparation process is shown in Figure 2.

**Table 3:** Latin name and family name of the herbs used in Vachaharidradi Gana Vati.

Serial No	Drug	Latin name	Family
1	Vacha	*Acorus calamus*, Linn	Araceae
2	Musta	*Cyperus Rotundus*, Linn	Cyperaceae
3	Devdaru	*Cedrus deodara* or *Pinus deodara*, Roxb	Pinaceae
4	Shunthi	*Zingiber officinale*, Roscoe	Zingiberaceae
5	Haritaki	*Terminalia chebula*, Retz	Combretaceae
6	Haridra	*Curcuma longa*, Linn	Zingiberaceae
7	Daruharidra	*Berberis aristata*, DC	Berberidaceae

The process of trituration is a part of the Ayurvedic pharmaceutical concept known as Bhavana. It enhances the bioavailability of the active compounds. Each tablet of Vachaharidradi Gana Vati contains approximately 50 mg of each herb, along with 5 mg of decoction of each herb. The quality control of the final products will be assessed by analyzing both the raw materials and final products of both drugs in a GMP-certified pharmacy.

### Criteria for Assessment

#### Objective Parameters

The anthropometrical parameters, weight, BMI, waist circumference, abdominal girth, mid-arm circumference, waist-hip ratio (WHR), and triceps skinfold thickness (TSFT), will be measured before and after treatment.

#### Instruments and Techniques Required for Anthropometric Data Collection

Children’s weight will be measured with an electronic portable scale with high sensitivity and specificity and will be calibrated from time to time. Height will be measured by portable stadiometers, with the participants’ feet placed together with heels, buttocks, and shoulder blades against the stick and head positioned in the Frankfurt horizontal plane. Waist and hip circumferences will be measured twice using inextensible anthropometric tape, with the children standing erect and relaxed with arms at the sides and feet positioned close together. Waist circumference will be measured midway between the lowest border of the rib cage and the upper border of the iliac crest, at the end of normal expiration [40].

Hip circumference will be measured at the widest part of the hip at the level of the greater trochanter. Mid-upper arm circumference will be taken at midpoint of the tip of the acromion process to the olecranon process, with the tape slung to the skin but not compressing the soft tissues. Both right and left sides will be measured, and the average will be taken [41].

For all measurements, the tape will be positioned at a level parallel to the floor. All measurements will be in centimeters to the nearest 0.1 cm, except TSFT. TSFT will be measured in millimeters at the midpoint between the tip of the acromion process and the olecranon process (back side of the middle upper arm) with the help of the calibrated skinfold caliper. BMI will be calculated as the ratio of body weight to the square of height (kg/m^2^), and WHR will be calculated as the ratio of waist to hip circumference [42]. Proper training will be provided to the personnel for accurate measurements using these techniques.

#### Investigations Proposed

Hemoglobin percentage, thyroid-stimulating hormone, and random blood sugar are the screening tests to be done only before treatment, whereas lipid profile will be done to assess the efficacy of interventions before treatment as well as after treatment. These laboratory tests will be performed using standard equipment and gold standard methods to ensure accurate results.

#### Subjective Parameters

Signs and symptoms of obesity (Sthaulya) are mentioned in the Ayurvedic texts, and for assessing the severity of each symptom, they were graded following the book called WHO-Direct Financial Cooperation sponsored project on Developing Guidelines for Clinical Research Methodology in Ayurveda. It is illustrated in Table 4 [43].

**Table 4:** Signs and symptoms of Sthaulya according to Ayurvedic classics.

Signs and symptoms or gradation	0	1	2	3	4
Pendulous hips, abdomen, breasts (chala sphika, udara, stana)	Absence of movements (in the areas) (Chalatva)	Little visible movement (in the areas) after rapid movement	Little visible movement (in the areas) after moderate movement	Movement (in the areas) after mild movement	Movement (in the areas) after changing posture
Bad odor (angagandha)	No odor	Bad odor but not offensive	Strong odor but can be lessened by the use of deodorants and perfumes	Very strong odor even after using fragrances (use of deodorants or perfumes)	—^a^
Perspiration (swedadhikya)	Sweating after heavy work and fast movement or in hot weather	Profuse sweating after moderate work and movement	Sweating after little work and movement (stepping ladder)	Profuse sweating after little work and movement	Sweating even at rest or in cold weather
Increased appetite (kshudha-adhikya)	As usual or routine	Slightly increased (1 meal extra with routine diet)	Moderately increased (2 meals extra with routine diet)	Markedly increased (3 meals extra with routine diet)	—
Increased thirst (pipasa-adhikya)	Feeling of thirst (7-9 times or 24 hours) and relieved by drinking water	Feeling of moderate thirst (>9-11 times or 24 hours) and relieved by drinking water	Feeling of excess thirst (>11 times or 24 hours) and not relieved by drinking water	Feeling of severe thirst (>13 times or 24 hours) and not relieved by drinking water	—
Exertional dyspnea (kshudra shwasa)	No dyspnea even after heavy work	Dyspnea after moderate work but relieved later and tolerable; dyspnea by climbing upstairs of 10 steps, and time taken will be more than 15 seconds	Dyspnea after little work but relieved later and tolerable; dyspnea by climbing upstairs of 10 steps, and time taken will be more than 25 seconds	Dyspnea after little work but relieved later and not tolerable; dyspnea by climbing upstairs of 10 steps, and time taken will be more than 35 seconds	Dyspnea in resting condition
Increased sleep (nidradhikya)	Normal and sound sleep for 6-8 hours or 24 hours with a feeling of lightness and relaxation in the body and mind	Sleep>8-9 hours with slight heaviness in the body	Sleep>9-10 hours or 24 hours with heaviness in the body associated with yawning (Jrimbha)	Sleep>10 or 24 hours with heaviness in the body associated with yawning (Jrimbha) and sleepiness (Tandra)	—

^a^Not applicable.

### Participants

It is crucial to calculate the effect size according to the equivalence or superiority formula with the help of the SD and mean difference of the reference paper [44]. This approach enhances the RCT, making it superior to other types of research. In this study, an attempt is made to assess the equivalence effect of Vachaharidradi Gana Vati against the standard controlled drug, Navak Guggulu. The equivalence formula is applied, and the sample size is calculated to minimize any bias. So, the formula for equivalence (parallel design) has been used to calculate the sample size of this study. For that, the reference values of the control drug have been used [44].

The formula for equivalence (parallel design) is:



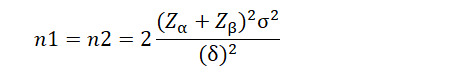



where *Z*_α_=1.96, α=type I error at 5%, σ=SD, *Z*_β_=0.84 (for equivalence 2-tailed), β=type II error at 20%, and δ=true differences in means.

Sample size (N) = *n*1 = 2 × (1.96 + 0.84)^2^ × (0.86)^2^/(0.57)^2^ = 39 each

Total=78 samples. With 10% dropout=43 each=86 samples.

### Justification on the Formula

The equivalence formula for parallel-group design is particularly suitable for this study, as it is specifically designed to determine the sample size needed to assess whether 2 interventions—Vachaharidradi Gana Vati and Navak Guggulu—are statistically equivalent within a defined margin of clinical acceptability. This approach is crucial for trials aimed at establishing equivalence rather than superiority or noninferiority. The formula accounts for the population variability in the population by incorporating the SD of the control drug and ensures a robust comparison by considering both type I and type II error rates, set at 5% and 20%, respectively. This balance between the risks of false-positive and false-negative results is critical in equivalence trials, where even small differences may have clinical significance.

By including the true difference in means (δ), the formula ensures that the calculated sample size is adequate to detect equivalence within the predefined margin. The adjustment for a 2-tailed test reflects the bidirectional nature of equivalence testing, ensuring that the study is powered to detect differences in either direction. Given these attributes, this formula provides a scientifically robust framework for calculating the required sample size, ensuring that the study’s conclusions about equivalence are statistically valid and clinically meaningful. The sample size ensures that each participant receives individualized care and monitoring.

For reference values, a clinical trial paper was used [44].

### Randomization and Allocation Sequence Generation

The earlier estimated sample size of 86 is increased to 88 children with overweight to have an equal balance in each group of the treatment.

#### Randomization Procedure for 88 Participants (44 Each Group)

For a total sample size of 88 overweight patients (44 in each group: Navak Guggulu and Vachaharidradi Gana Vati), block randomization will be used to ensure balanced allocation throughout the study [45]. A block size of 4 will be used, dividing the sample size into 22 blocks. Each block will consist of predefined sequences, such as AABB, ABAB, BABA, or BBAA, where “A” represents group A (Navak Guggulu) and “B” represents group B (Vachaharidradi Gana Vati). These sequences ensure that within each block, participants are equally allocated to both groups. The randomization sequence will be generated using a computerized random number generator or statistical software, ensuring variability in block order. The randomization process will be managed by a trained, unblinded departmental faculty member who is not involved in the study. The block randomization procedure ensures that both groups maintain equal sample sizes while preventing predictability, preserving the study’s internal validity and robust design.

#### Allocation Implementation

To maintain allocation concealment, the group assignments will be placed in SNOSE, with each envelope containing 1 allocation based on the pregenerated sequence. The SNOSE technique is widely used in clinical trials [46]. The process of assigning treatments to these envelopes will be conducted by personnel not involved in enrolling or assessing participants. To maintain blinding, treatments will be labeled with coded identifiers. Treatments A and B will be recoded by unblinded, trained faculty using either code III or code IIII. This unblinded faculty will not be involved in the trial’s conduct and assessment. The blind code III or IIII will be labeled on the respective patient numbers on the opaque containers by the trained, unblinded faculty. These recoded opaque containers will then be handed over to the investigator for further administration to the patients. An unblinded, trained faculty will also provide 88 sealed envelopes to the investigator, containing unblinded information about the treatment administered to each patient. These sealed envelopes will be kept intact under the investigator’s custody until the completion of the last visit of the last enrolled patient in the study and stored in a manner that prevents accidental disclosure. A master chart will be prepared as a data source for statistical analysis and will be handled with high security, ensuring confidentiality and limited access to authorized personnel only. When the data have been locked, the outcome assessor (statistician) will unblind the treatment codes for statistical analysis.

### Data Collection Tools and Process

This study will use Ayurveda Samhitas (textbooks on Ayurveda), modern texts, and a web-based search of PubMed, Google Scholar, etc. Navak Guggulu (tablet) and Vachaharidradi Gana Vati (tablet) will be prepared in a GMP-certified pharmacy. The study will also use a case record form, patient information sheet, written dietary restriction, and written informed consent form. The observers will be trained properly to assess the subjective parameters and anthropometrical measurements to ensure proper data collection in the case record Form. The periodic follow-up of every fortnight for 90 days has been advocated, so the data of objective measurements and subjective criteria will be collected in case record forms every fortnight to ensure adherence. The data regarding lipid profile values will be collected before and after intervention. Quality checks will be performed manually.

### Outcomes

#### Primary Outcome—BMI

BMI will be calculated as the ratio of body weight to the square of height (kg/m^2^). Indian Academy of Pediatrics 2015 charts will be used to categorize weight as normal, overweight, and obese.

#### Secondary Outcome

The secondary outcomes are as follows: (1) anthropometric measurements including mid-upper arm circumference, abdominal girth, waist circumference, hip circumference, and WHR in children with overweight; (2) reduction in lipid profile values in children with overweight; and (3) reduction of signs and symptoms of obesity (Sthaulya) mentioned in Ayurvedic classics following the gradations in the book called WHO-Direct Financial Cooperation sponsored project on Developing Guidelines for Clinical Research Methodology in Ayurveda [43].

### Statistical Analysis

The statistical analysis will be performed at the end of the study. It will involve various tests to evaluate both within-group and between-group efficacy of Navak Guggulu and Vachaharidradi Gana Vati in managing Sthaulya (overweight) among children. For normally distributed continuous variables like BMI, WHR, and skinfold thickness, paired 2-tailed *t* tests will be used to evaluate the efficacy of each intervention within the same group (pre- and posttreatment comparisons).

For nonnormally distributed data, the Wilcoxon signed rank test will be used. For normally distributed data, independent 2-tailed *t* tests will be used for between-group comparisons of mean changes; for nonnormal data, the Mann-Whitney *U* test will be used.

To ensure valid comparisons, the analysis of covariance will be used to account for any potential baseline variations between groups. To evaluate residual efficacy after treatment discontinuation at 180 days, repeated measures ANOVA will analyze longitudinal changes in outcomes, while mixed-effects models will handle missing data or variability across time points. For stratified analysis based on Desha (geography), Kala (season), and Prakruti (body constitution), multivariate regression analysis will identify predictors of treatment efficacy, and interaction analysis will explore the influence of these factors. A statistician consultation will be considered for the selection of the proper statistical method.

### Data Monitoring Committee

It consists of the researcher, under the guidance of the supervisor and cosupervisor, along with the statistician and departmental faculty members. The progress will be monitored by supervisors and statisticians by reviewing the group-wise case record forms and master charts.

### Ethical Considerations

The Mahatma Gandhi Ayurved College Hospital and Research Centre’s Institutional Ethics Committee approved this study (registration: MGACHRC/IEC/July-2022/521), and it was also registered with the Clinical Trial Registry of India (CTRI/2023/03/051135). The same ethical committee also approved the addition of the site at CSMSS Ayurved College, Chhatrapati Sambhajinagar. After receiving a No Objection Certificate from the institutional ethical committee of the CSMSS Ayurved College (AMCS/1407/2023), the participant recruitment at both sites will be initiated. The institutional ethical committee will decide on the end point and oversee the trial as it progresses. The researchers will assess any adverse events and will report to the ethics committee. Consent from parents and assent from patients (older than 12 years) will be obtained before conducting the trial in the local language while explaining every aspect of the study. Every participant will get information regarding the study’s design and objectives, data confidentiality, and the freedom to withdraw from the study at any moment without giving a reason. The informed consent form includes a patient information sheet (part 1) and a certificate of consent (part 2). Secondary analysis will be covered without additional consent. Data will be anonymized, and the personal information of the participants will be kept confidential before, during, and after the trial. Physical data will be stored in a protected storage facility, with access available only to the researchers. Computerized data will be held in a password-protected computer. Data access will be restricted to members of the research team only. During data analysis, measures will be taken to protect participants’ privacy. Participation in the study is completely voluntary; hence, no incentive will be provided to the participants. During the treatment period, if the patients’ condition worsens, the case will be excluded and will receive conventional treatment and care until the symptoms subside, free of charge. The ethics committee will be informed of the patient’s condition. The therapy will be discontinued, and the child will be withdrawn from the study if any other illness arises during the treatment period that could influence study outcomes. Similarly, if a participant experiences personal issues that prevent continuation of therapy and are likely to affect the study results, they will also be withdrawn. If the patient misses any of the periodic follow-ups during the intervention, they will be excluded from the study.

## Results

In April 2024, the randomized controlled study got underway. Since participants may be in the study at any time, our goal is to complete the research by May 2025. Any significant changes to the protocol will be posted on ClinicalTrials.gov. The study’s findings will be disseminated through publications.

## Discussion

### Principal Findings

Childhood obesity has been rising in the past 3 decades. Its prevalence is increasing both in the higher socioeconomic groups as well as lower socioeconomic groups [7]. The short- and long-term associations between overweight and a range of adverse health-related outcomes are well established and raise the level of importance for understanding overweight as a major public health concern for children and adolescents. Lack of physical activities and uncontrolled dietary habits are the root causes of higher incidence rates of overweight in children. Nowadays, increased screen time is also contributing to this problem. It increases motivated responses to food intake and snacking behavior among children and adolescents [47,48].

Vachaharidradi Gana is a herbal compound mentioned in Ayurvedic classics that is effective in the reduction of excessive fat by its Kaphamedohara property. In the case of Sthaulya [49], there is an abundance increase in Kapha and Medo Dhatu (fats). These Dhatu are predominant in Jala (water element) and Pruthvi (earth element) Mahabhuta. The contents of the Vachaharidradi Gana are opposite in characteristics to that of Kapha-Medodhatu. Thus, it is expected to break the etiopathogenesis of Sthaulya if administered to children with overweight.

Dietary changes are directly linked to weight loss. Fixed lunch and dinner times, low-fat meals, fiber-rich vegetables, and reduced frequency and amount of food are advocated in the management of Sthaulya [50]. However, it may be a matter of concern to maintain the reduced weight for a longer period. Hence, with the addition of diet control and medicine administration, it is essential to add some behavioral changes too. Behavioral modifications are found to be useful for good weight maintenance. In Ayurveda, it is aimed at exercises or yogic practice. Yogic practices involve stretching and relaxation of various muscles steadily, which breaks down triglycerides and mobilizes fatty acids. Yogic practices also act synergistically with diet restrictions in Sthaulya [51,52]. Yoga practices such as Suryanamaskara can be an enjoyable and engaging way to encourage physical activity among children, fostering self-discipline, which could extend to healthier eating habits and more structured activity levels with improved mental well-being. While specific studies on Suryanamaskara for childhood obesity are limited, studies on general yoga practices for children have shown promising results in reduced BMI and waist circumference and are equally effective as the standard weight management for BMI reduction in the pediatric and adolescent age group [53-55]. One-hour yoga session for 12 weeks comprising Suryanamaskara, asanas, pranayama, and meditation in adolescent girls having polycystic ovarian syndrome showed significant alterations in blood glucose, insulin, and lipid levels [56]. A review paper on Suryanamaskara concluded that if practicing Suryanamaskara is initiated in children at the age of 7-8 years, it helps them grow better not only physically but also with great mental health [36]. In this study, the daily performance of Suryanamaskara is advocated in the form of a yogic practice. Hence, it was hypothesized that the Vachaharidradi Gana Vati along with diet restriction and Suryanamaskara will help in the reduction of the weight and BMI of children with overweight.

Navak Guggulu, on the other hand, is a control drug, whose efficacy has been proven in obesity and hyperlipidemia. The addition of diet restriction and Suryanamaskara will add further to its proven efficacy. The effect of combined interventions in both groups is expected for a sustained period even after the stoppage of the medicines.

This is the first study in which Suryanamaskara is advocated as part of a multidimensional treatment modality for reducing weight and BMI in children. It will further elaborate on the benefits of Suryanamaskara in children. It is expected that this modality will also help to lower the raised values (if any) of lipid profiles and the signs and symptoms of Sthaulya.

Children do not tolerate dosage that is in powdered form; therefore, changing the powder form of a medication into a tablet makes dosage calculations simple and makes sure that the recommended quantity is administered. Further, the double blinding will help in controlling performance bias and ascertainment bias that might inadvertently sweep into the study.

Being overweight deteriorates cognitive functioning in children while it adversely affects them psychologically. Children with overweight show behavioral disorders, a high emotional nature, damaged self-awareness, and low self-evaluation and demonstrate unhappiness. It is a cause of social concern [57,58].

Considering all these factors, treating children with overweight and preventing them from becoming obese are of utmost importance. The available research on overweight in children is limited. Given the multifactorial etiopathogenesis of children with overweight, drug administration alone will not be sufficient for treatment. Therefore, a multidimensional approach to treating children with overweight will be practiced in this study. Positive study findings may implicate a feasible, promising, noninvasive, and affordable intervention for managing children with overweight, which also offers sustainable holistic lifestyle modifications and positive psychological changes. This study could enhance the scientific credibility of Ayurveda in managing childhood obesity and support its integration into modern health care guidelines. The use of locally available, affordable herbs like Vachaharidradi Gana Vati could make obesity management more accessible in low-income settings.

### Limitations

The parents may resist accepting the fact that their children with overweight need treatment. It may be difficult to monitor whether the children follow diet restrictions and Suryanamaskara for a longer period. Additionally, as this is a PhD study without external funding, the sample size is relatively small.

### Potential Avenues or Strengths of Study

Ayurveda herbal medicines have great potential or impact to cure the disease naturally without any side effects. The Ayurvedic diet and lifestyle recommendations can be added as multidimensional aspects of the conventional treatment modality.

This research trial is planned to know the Kaphamedohara effect of the classical formulation by administering it orally to children with overweight. The positive outcome of the study will facilitate the commercialization of the product, Vachaharidradi Gana Vati. Further, the medicines used in the study can be patented and made copyrighted later and publicized for the benefit of the population. Large-scale, multicentric RCTs can be conducted to test the effect of Vachaharidradi Gana Vati on children with overweight and also children with obesity. Such a trial can be conducted in adults and young children (aged 3-9 years) as well. Longitudinal studies could evaluate the long-term impact of Ayurvedic interventions on childhood obesity, focusing on not only weight and BMI management but also quality of life, psychological well-being, and prevention of obesity-related diseases in adulthood.

### Conclusions

This research will be useful to provide new insight into the effectiveness of the Ayurveda pharmacological approach in treating obesity in children. The results will guide evidence-based multimodality in treatments aimed at changing lifestyle behaviors in children with obesity, thus reducing social stigmatization in them.

## Abbreviations

CSMSS: Chhatrapati Shahu Maharaj Shikshan Sanstha
GMP: Good Manufacturing Practice
RCT: randomized controlled trial
SNOSE: sequentially numbered, opaque, sealed envelope
SPIRIT: Standard Protocol Items: Recommendation for Interventional Trials
TSFT: triceps skinfold thickness
WHO: World Health Organization
WHR: waist-hip ratio

## Data Availability

The study is ongoing, and the datasets that will be generated and analyzed will be available from the corresponding author upon reasonable request.
